# Using biologically synthesized TiO_2_ nanoparticles as potential remedy against multiple drug resistant *Staphylococcus aureus* of bovine mastitis

**DOI:** 10.1038/s41598-023-45762-4

**Published:** 2023-11-01

**Authors:** Anwar Ul-Hamid, Nadeem Baig, Ali Haider, Abbas S. Hakeem, Muhammad Ikram

**Affiliations:** 1 Core Research Facilities, King Fahd University of Petroleum and Minerals, 31261 Dhahran, Saudi Arabia; 2 Interdisciplinary Research Center for Advanced Materials, King Fahd University of Petroleum and Minerals, 31261 Dhahran, Saudi Arabia; 3 Interdisciplinary Research Center for Membranes and Water Security, King Fahd University of Petroleum and Minerals, 31261 Dhahran, Saudi Arabia; 4 Department of Clinical Sciences, Faculty of Veterinary and Animal Sciences, Muhammad Nawaz Shareef University of Agriculture (MNSUA), Multan, 66000 Pakistan; 5 Interdisciplinary Research Center for Hydrogen and Energy Storage, King Fahd University of Petroleum and Minerals, 31261 Dhahran, Saudi Arabia; 6 Solar Cell Applications Research Lab, Department of Physics, Government College University, Lahore, 54000 Pakistan

**Keywords:** Materials science, Nanoscience and technology

## Abstract

Presently, there is considerable emphasis on biological synthesis of nanoparticles containing bioactive reducing compounds with an aim to mitigate the harmful effects of pollutants. The approach under study is simple and ideal for the production of durable antimicrobial nanomaterials by novel single-step green synthesis of TiO_2_ metal oxide nanostructures using ginger and garlic crude aqueous extracts with bactericidal and catalytic activity. A variety of experimental techniques were used to characterize the synthesized nanomaterials. As demonstrated using x-ray diffraction and ultra-violet visible spectroscopy, the produced nanoparticles exhibited high absorption at 318 nm with size varying between 23.38 nm for ginger and 58.64 nm for garlic in biologically-reduced TiO_2_. At increasing concentrations (500, 1000 µg/50 µl), nanoparticles reduced with garlic exhibited enhanced bactericidal efficacy against multiple drug-resistant *S. aureus* and effectively decomposed toxic methylene blue (MB) dye. In conclusion, biologically-reduced TiO_2_ nanoparticles may prove an effective tool in the fight against microbial illnesses and drug resistance.

## Introduction

Mastitis is commonly caused by bacteria and exhibits several etiologies^1^. Globally, bovine mastitis (BM) is the leading cause of financial loss in milk industry^2^. Although mastitis may be triggered by 137 distinct bacteria^3^, *Staphylococcus aureus* is most typically associated with this condition and is usually linked to subclinical or persistent infections. The recovery rate of antibiotic therapy for this agent is poor, hence, the illness has not been adequately eradicated and/or managed in several herds^4,5^. This could be linked to the ability of *S. aureus* to build biofilm, live inside epithelium and macrophages, and withstand antibiotic medication^6–8^. To manage BM, antibiotics have become the most prevalent method to manage BM, however medicines such as chloramphenicol, ciprofloxacin, novobiocin, vancomycin, and tetracycline have been documented to be ineffective against *S. aureus*^9–11^. Multiple antibiotics have been exploited in the treatment of BM, nevertheless treatment failure is now often documented^12^. The purposeful administration of antimicrobial drugs in veterinary treatment is a significant contributor towards transmission of antibiotic-resistant bacterial infections to human populations, hence posing a public health risk^12,13^. According to reports, methicillin-resistant *Staphylococcus aureus* (MRSA) poses a concern to both public and animal health. Multidrug-resistant (MDR) *S. aureus* infections have been linked to substantial morbidity and economic losses^14–16^. *S. aureus* is often detected in dairy cow's milk and has been scientifically confirmed to be a mastitis-causing agent^17^. According to reports, humans also ingest raw milk occasionally^18^. Currently, livestock-associated MRSA and community-associated MRSA have been described and constitute a major public health safety issue^19–22^. *S. aureus* infections have reportedly been linked to the intake of tainted milk and its products^23^ which, often cause septicemia, pneumonia, and dermatitis in humans^24^.

Nanoparticles (NPs) are often classified as entities between 1 and 100 nm in size. These particles are intriguing because their physicochemical characteristics vary significantly from those of their macroscale counterparts^25^. Metal oxide nanoparticles are most frequently exploited nanostructures, based on the number of NPs generated annually by the industrial sector. TiO_2_, ZnO, and SiO_2_ are widely produced NPs. Particularly, global TiO_2_ production has surpassed 10,000 tons per annum^26^. TiO_2_ nanoparticles have several uses in numerous domains, including photocatalysis^27^, sensors^28^, and antibacterial agents^29^. It is a useful semiconducting transitioning metal oxide material that exhibits particular characteristics such as simple control, low cost, non-toxicity, and strong resistance to chemical erosion; thus, it is used in solar cells, chemical sensors, and environmental desalination^30,31^. These nanoparticles exhibit unique electrical, magnetic, and optical capabilities compared to their bulk counterparts. TiO_2_ may exist in amorphous and crystalline forms, with anatase, rutile, and brookite being the most common crystalline polymorphs^32^.

Ginger, also known as the bulb of *Zingiber officinale*, is native to tropical Asia. The plant's rhizomes are widely used as a spice, flavoring, or aroma in foods, drinks, soaps, cosmetics, and most importantly in medicine. It contains antioxidant and bactericidal characteristics and is used to treat stomach pain, cough, and also lowers the intensity of transient chemotherapy-induced nausea in young cancer patients^33,34^.

Garlic (*Allium sativum*), extensively used in medicine as an antibiotic, serves as a renowned natural ingredient used in green synthesis. It is composed of allicin (76%) (diallyl thiosulphinate), methyl allyl thiosulphinate (5–7%), allyl methyl thiosulphinate (14.5%), and dimethyl thiosulphinates (2.6%)^35^. Garlic's bactericidal effect is often linked to the constituent allicin, which contains sulfhydryl-modifying action and may thus block sulfhydryl-containing enzymes^36^. Allicin has also been shown to hinder RNA synthesis in microorganisms^37^ and lipid production, resulting in cell wall disruption^38^. In addition, it has broad antibacterial action against both gram-negative and positive microbes^39^. Garlic antibacterial action has been linked to the radical scavenging ability of its organo-sulfur compounds^40^. Its tendency to produce free radicals is also a contributing factor towards its antibacterial activity^41^.

Nanoparticle production can be divided into two broad categories: bottom-up and top-down approaches. The atom, cluster, and nanoparticle are progressively built up in bottom-up or constructive procedure. Spinning (involves a chemical solution serving as precursor to a combined system of distinct fragments) and sol–gel (involves chemical mixture serving as precursor to an interconnected network of distinct particles) are two examples of wet-chemical processes that allow for the regulation of physical characteristics. Nitrogen or additional inert gases are often used to purge the accelerator of oxygen and prevent chemical processes^42^. The prelude and water are injected into the disc as it rotates at a distinct speed. Fusion of atoms or molecules form precipitates, which are lumped together and dried due to the act of spinning^43^, in chemical vapor deposition (CVD) chemical response takes place where an exploded material comes into contact with merged gas^44^), whereas pyrolysis includes burning a precursor using flame. The precursor could be a liquid or vapor, and is introduced inside the furnace via a tiny hole under high pressure before being burned^45^. In order to retrieve nanoparticles from combustibles or by-product gases, bottom-up approaches such as biosynthesis (which employs bacteria, herbal extracts, fungi, etc. together with precursors to generate the nanoparticle rather than conventional chemicals for bio-reduction and capping) and air classification are widely used. The top-down approach involves breaking a substance down to its atomic building blocks such as milling and subsequent annealing of nanoparticles in an inert environment^46^, nanolithography (constitutes printing an essential construct on a light-sensitive matter by deliberately eliminating an area of material to generate the intended shape), laser ablation (the illumination of metal immersed in water solution due to laser beam compressing a plasma plume that protrudes from the exterior of metal), and thermal degradation (an endothermic chemical breakdown induced by heat which destroys chemical connections in the substance^47^) and sputtering (a formation of thin film of nanoparticles accompanied by annealing are common steps in the sputtering process). Among the most popular approaches to create nanoparticles is application of thermal breakdown (when an element is heated to its own unique disintegration temperature). Various diverse methods such as plasma enhanced chemical vapor deposition (PE-CVD), in situ fabrication, sol–gel procedure, solid state techniques^48–50^ and biosynthesis, are used to produce TiO_2_ nanoparticles. Among these approaches, biosynthesis of TiO_2_ nanoparticles has attracted the most interest owing to its simplicity, nontoxicity, and cost-effectiveness, etc. Various plant components (roots, stem, leaf, flower, peel) act as oxidizing, reducing, and capping substances and are employed to modulate NPs' formation and agglomeration^51^. The comparative analysis with published literature is depicted in Table S1.

BM has emerged as a threat to the farming sector. The prevalence and resilience of *S. aureus* across microbes is on the rise, suggesting that microbes are a significant source of infection. The recent development of antibiotic resistance is an important concern for public health. Curing illnesses and maximizing safe milk yield by the use of antibiotics will no longer be acceptable in the future. Due to these issues, antibiotic overuse must be reduced, and substitute therapeutic methods must be implemented. New antibacterial substances are being developed due to breakthroughs in nanobiotechnology, particularly the ability to produce metal oxide nanomaterials of specified form and dimension. The current investigation is aimed at evaluating the antibacterial properties and catalytic effectiveness of single-step green-produced low-cost, non-toxic and unique TiO_2_ nanostructures using ginger and garlic CAE to combat MDR *S. aureus* isolates of bovine mastitis, that results in enormous production decline in dairy sector and adverse effects on public health and economy due to resistant subclinical or persistent infections in humans and animals. This investigation is unique from veterinary standpoint since it uses cost-effective and eco-friendly synthesis approach and evidential molecular docking analysis.

## Materials and methods

### Materials

Titanium ethoxide Ti_4_(OCH_2_CH_3_)_16_ and sodium hydroxide (NaOH) and DPPH (2,2-diphenyl-1-picryl-hydrazyl-hydrate) of scientific grade were obtained from Sigma Aldrich for green fabrication of TiO_2_ nanostructures. Ginger and garlic roots were procured from a local market. All additional chemicals and growth medium used were of laboratory grade. Collection of ginger and garlic roots material complied with our institutional, national, and international guidelines and legislation.

### Crude aqueous roots extract (CAE) preparation

Ginger and garlic root flour was produced using an electric mill and stored in acrylic flasks after the recovery of fine dust. The resulting root powder was mixed with a regulated amount of DI water (1:10) and agitated at 70 ℃ for thirty (30) minutes using a magnetic stirrer. After filtering, the generated extracts were filtered using Whatman No. 1 paper. The resulting filtrate was refrigerated at 4 ℃ until subsequent processing^52^.

### Green synthesis of TiO_2_ nanostructures

**Figure 1:**
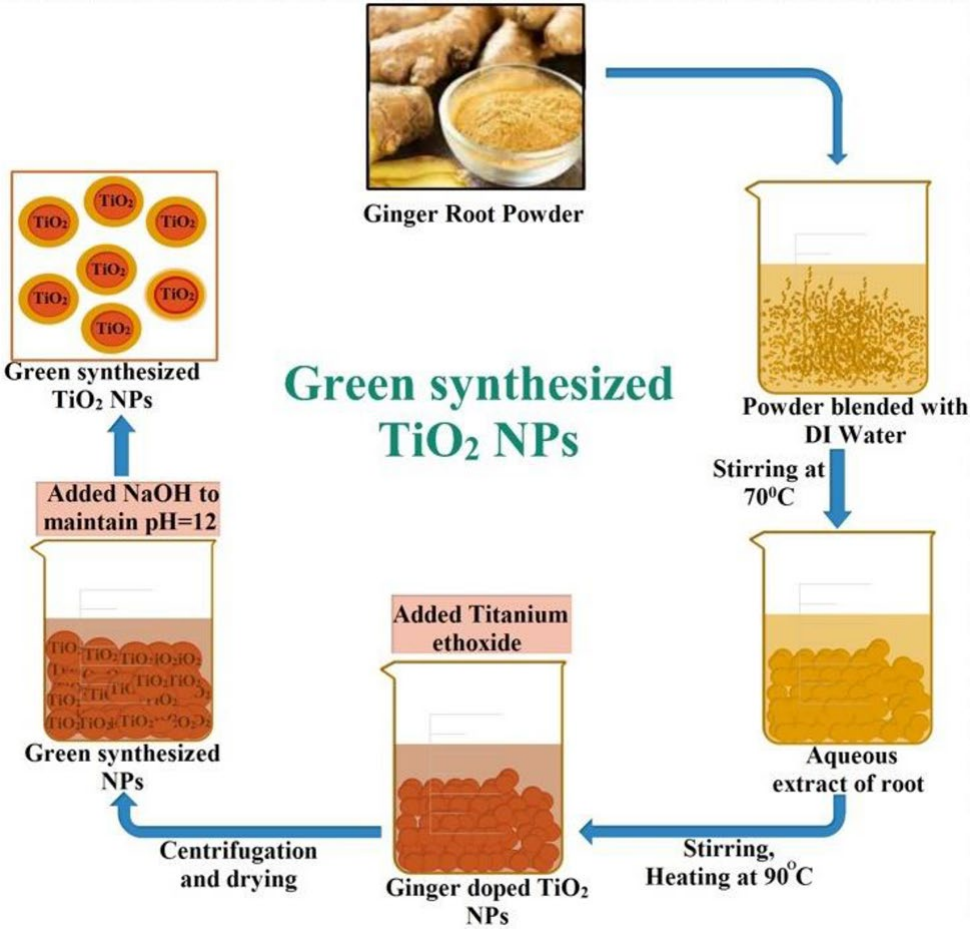
Schematic illustration of biologically fabricated TiO_2_-NPs.

Numerous proportions of ginger and garlic CAE (1.2, 1.8, 2.4, 3.0, 3.6 and 4.2 ml) were applied to 0.1 M (50 mL) titanium ethoxide Ti_4_(OCH_2_CH_3_)_16_ with stirring. After achieving the appropriate pH (12) with the inclusion of 2.0 M sodium hydroxide, the mixture was stirred for two hours at 90 °C till precipitates formed. The pellet was produced after 10 min of centrifugation at 10,000 rpm and washed with deionized water (DIW) followed by drying overnight in a hot air oven at 90 °C. Different ratios of CAE in salt solution (1.2 ml:1, 1.8 ml:1, 2.4 ml:1, 3.0 ml:1, 3.6 ml:1 and 4.2 ml:1) were used to optimize green nanostructures as depicted in Fig. 1^53^.

### Characterization

In order to measure absorbance (ƛ_max_), the synthesized NPs were evaluated with UV–visible spectrophotometer across the wavelength ranges of 250–800 nm. Employing a BRUKER D2 Phaser (XRD) with a 2θ range (10°–80°) and Cu K_α_ radiation of = 1.540 Å, the composition and structure of loaded nanostructures were examined. Fourier-transform infrared spectroscopy (FTIR) was employed to evaluate the functionality of finished product. The elemental content of generated metal oxide nanostructures was examined using energy-dispersive X-ray spectroscopy (EDS). JEOL FE-SEM and JEM-2100F TEM instruments were deployed to validate the size and shape of produced nanostructures. The sample frame and concomitant band gap analysis were explored with X-ray photoelectron spectroscopy (XPS).

### Acquisition and characterization of multiple drug resistant *S. aureus*

#### Specimen collection

Specimens of mastitic fluid were acquired from clinically positive cattle at a number of local and state veterinary facilities and dairies in Punjab, Pakistan. The samples were purified by growing in order to check for microorganisms. All experimental protocols were approved by Muhammad Nawaz Shareef University of Agriculture (MNSUA) Multan, Pakistan. All methods were carried out in accordance with relevant guidelines and regulations and reported in accordance with ARRIVE guidelines.

#### Isolation of multiple drug resistant *S. aureus*

The cattle milk specimens were maintained on 5% sheep blood agar (SBA) and stored at 37 °C for 24–48 h^54^. The acquired benchmark colonies were striped in triplicate on Mannitol Salt Agar to purify *S. aureus*. Employing the guidelines of the National Committee for Clinical Laboratory Standards (NCCLS) for separation of multidrug-resistant *S. aureus*, the susceptibility of characteristic isolates to specified antibiotics was evaluated through disc diffusion. Microbicidal discs containing Oxytetracycline (30 μg), Tylosine (30 μg), Gentamicin (10 μg), Ciprofloxacin (5 μg), and Trimethoprim + Sulfamethoxazole (1.25 μg + 23.75 μg) procured from Bioanalyse® (Turkey) were coated to Mueller–Hinton agar (MHA). Bacteria exhibiting resistance to at least three antibiotics were declared as multiple drug resistant (MDR)^55^ after overnight incubation at 37 °C. Microbial colonies were classified based on visual characteristics using Gram's stain and biochemically with coagulase and catalase tests in accordance with Burgey's Manual of Determinative Bacteriology^56^.

### In-vitro bactericidal evaluation

In-vitro antibacterial assessment of green metallic oxide nanostructures was conducted upon prevalent bovine mastitogen, MDR *S. aureus* isolates. A total of 10 MDR *S. aureus* field isolates were collected. Using well diffusion technique, the in-vitro microbicidal activity was measured. The petri plates were swabbed with 0.5 Mc-Farland standard culture of *S. aureus* on MSA^55^. A sterilized cork borer was used to form 6-mm-diameter bores. Various proportions of CAE of ginger and garlic were infused as (10 mg and 50 mg/100 µl) and TiO_2_ at values of 0.5 and 1.0 mg/50 µl. Positive reference was Ciprofloxacin (0.005 mg/50 µl) while neutral control was DIW (50 µl). Following an overnight incubation at 37 °C, the microbicidal effectiveness was determined by measuring inhibition areas using a Vernier caliper.

#### Statistical analysis

The inhibition regions were evaluated using SPSS 20.0 with one-way variance test containing significance threshold of 5%,

### Radical scavenging assay (DPPH)

The anti-oxidant efficacy and free radical scavenging capacity of synthesized nanostructures were studied using a modified version of DPPH scavenging assay. For both ginger and garlic, a 0.1 mM DPPH solution was infused with TiO_2_ nanoparticles at concentrations ranging from 50 to 200 µg/mL. This was then vortexed and allowed to incubate in the dark for 30 min at ambient temperature. Ascorbic acid reference sample solutions were used. Each sample's scavenging rate (%) was determined using degradation of DPPH solution at 517 nm via corresponding Eq. (1).

1 $${\text{Scavenging}}\,{\text{rate}}\,\left( \% \right) = {\text{A}}_{0} - {\text{A1}}/{\text{A}}_{0} \times {1}00$$

A_0,_ A_1_ = Control absorbance, standard absorbance.

### Molecular docking analysis

**Figure 2:**
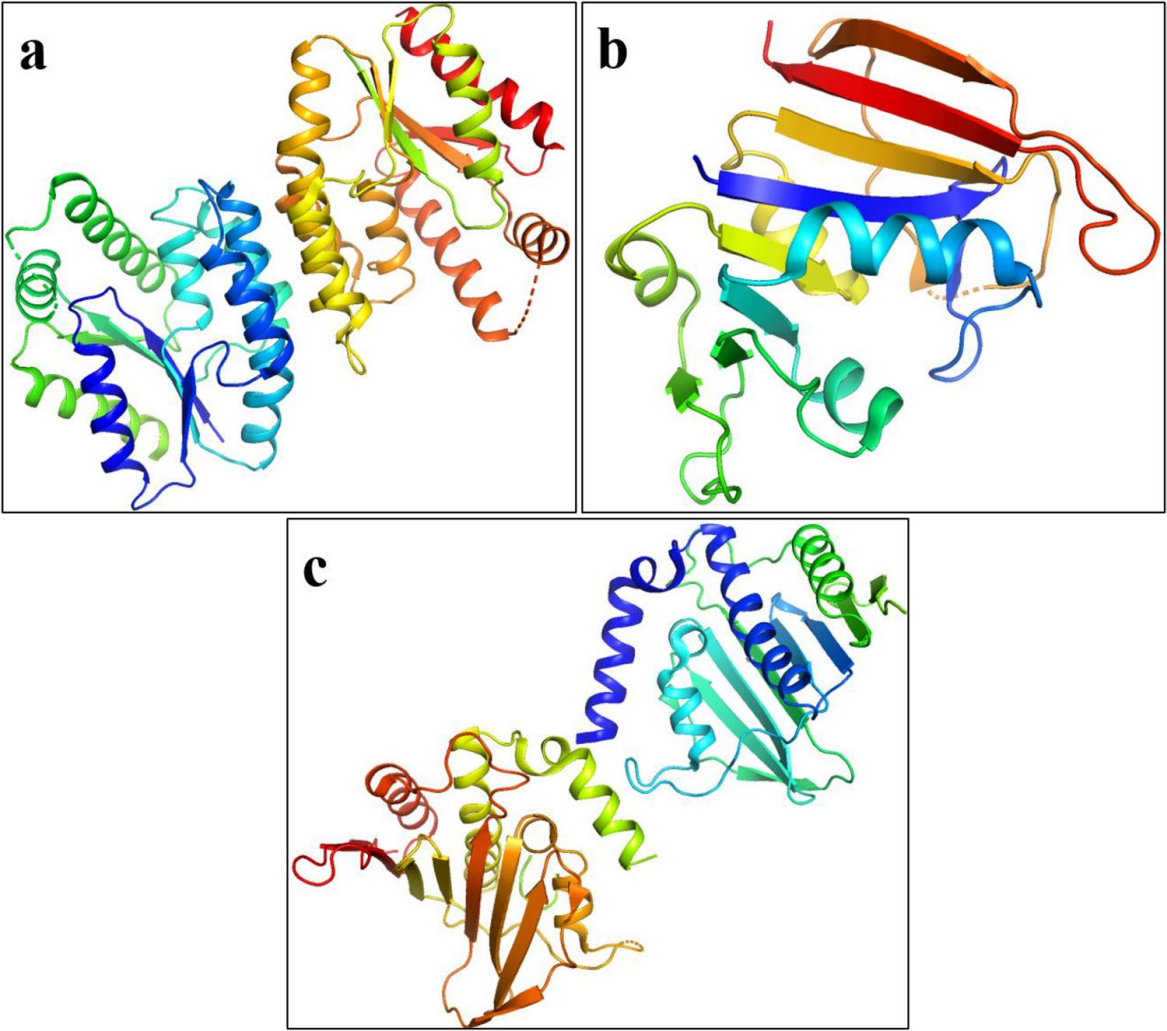
3D-structure of target proteins of *S. aureus*, (**a**) Thymidylate kinase (PDB: 4HEJ), (**b**) DNA gyrase (PDB: 5CTU), (**c**) Dihydrofolate reductase (PDB: 3FY8).

**Figure 3:**
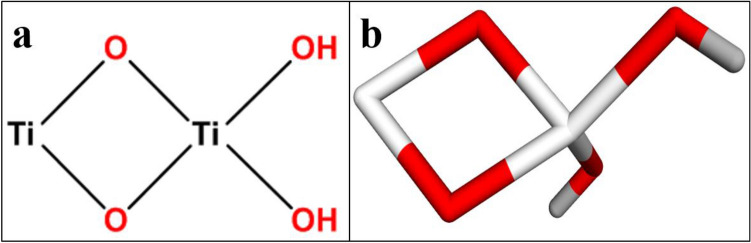
Structure of TiO_2_ nanostructures in (**a**) 2D and (**b**) 3D view.

TiO_2_ nanostructures were submitted to molecular docking analysis for determination of their binding interactions with potential substrates that are critical for bacterial cell proliferation. The enzymes engaged in DNA formation and folic acid production are well-known, desirable, and realistic targets for identification of antibiotics. Several protein targets, including dihydrofolate reductase (DHFR), Thymidylate kinase (TMK), and DNA Gyrase, were chosen for molecular interaction study. The 3D-structures of dihydrofolate reductase (PDB ID: 3FY8), resolution:2.20 Å^57^, thymidylate kinase (PDB ID: 4HEJ), resolution:2.00 Å^58^, and DNA gyrase (PDB ID: 5CTU), resolution:1.45 Å^59^, were obtained from protein database bank Fig. 2a–c. The program *SYBYL-X 2.0* was used for docking analysis^60^ *Sybyl-X2.0*/SKETCH module was used to generate 3D structures of selected compounds as illustrated in Fig. 3^61^, accompanied by energy reduction pursuant to the Tripos force field with Gasteigere Hückel atomic charge^62^. Flexible molecular docking simulations were conducted using Surflex-Dock module of molecular modelling software program *SYBYL-X 2.0*^62^. To study the binding affinities of nanoparticles with the active region residues of chosen proteins, required hydrogens were added. Atom kinds and atomic charges were determined based on the AMBER 7 FF99 force field. To avoid steric conflicts, the energy was reduced using Powell method with a convergence rate of 0.5 kcal/(mol・A) over 1000 cycles. At least 20 optimal docking positions were preserved for every ligand–receptor complex system. Using the Hammerhead scoring function, top potential positions of ligands were ranked. Surflex dock module utilizes an empirically determined consensus scoring (cScore)^63^ function which combines Hammerhead's empirical scoring function^59^, that is, D-score (dock score), G-score (gold score), Chem-score, potential mean force (PMF) score, and the total score, with a molecular resemblance technique (morphological identity) to create and evaluate hypothetical poses of ligand fragments^64^.

### Catalytic activity

**Figure 4:**
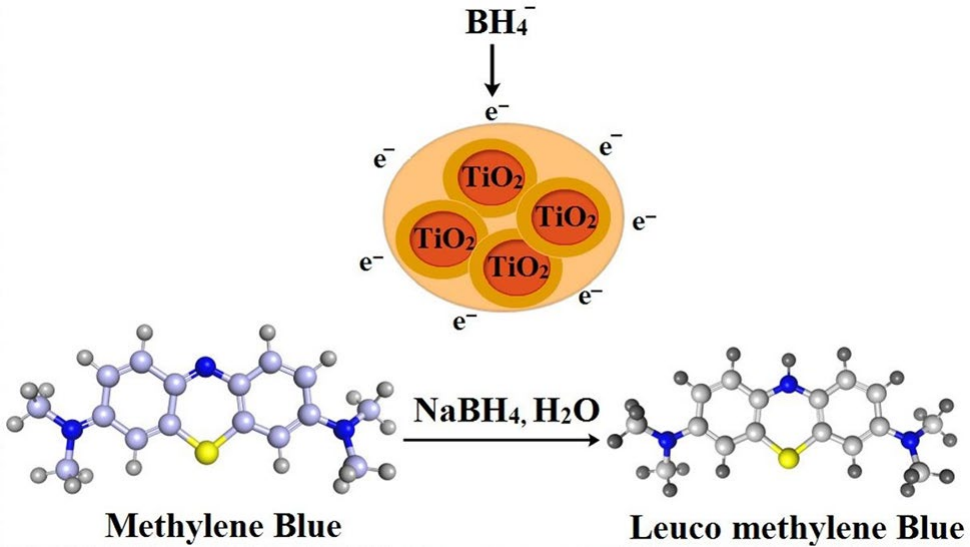
Diagrammatic representation for catalytic reduction of MB to LMB by green-synthesized NPs.

For catalytic evaluation of produced TiO_2_ nanostructures, 3 mL of methylene blue (0.03 × 10^−3^ M) and 300 µL of newly produced sodium boro-hydride hydro solution were mixed. The ideal concentration (3600 µL:1) of the material at a concentration of 6.0 mg/300 µL was then administered to the solutions. As a consequence, the methylene blue (MB) dye pigment disappeared, as seen in Fig. 4, indicating a degradation of the dye to leuco methylene blue (LMB). The UV–Vis spectrophotometer was employed to determine absorption within the range of 200 to 800 nm.

## Results

**Figure 5:**
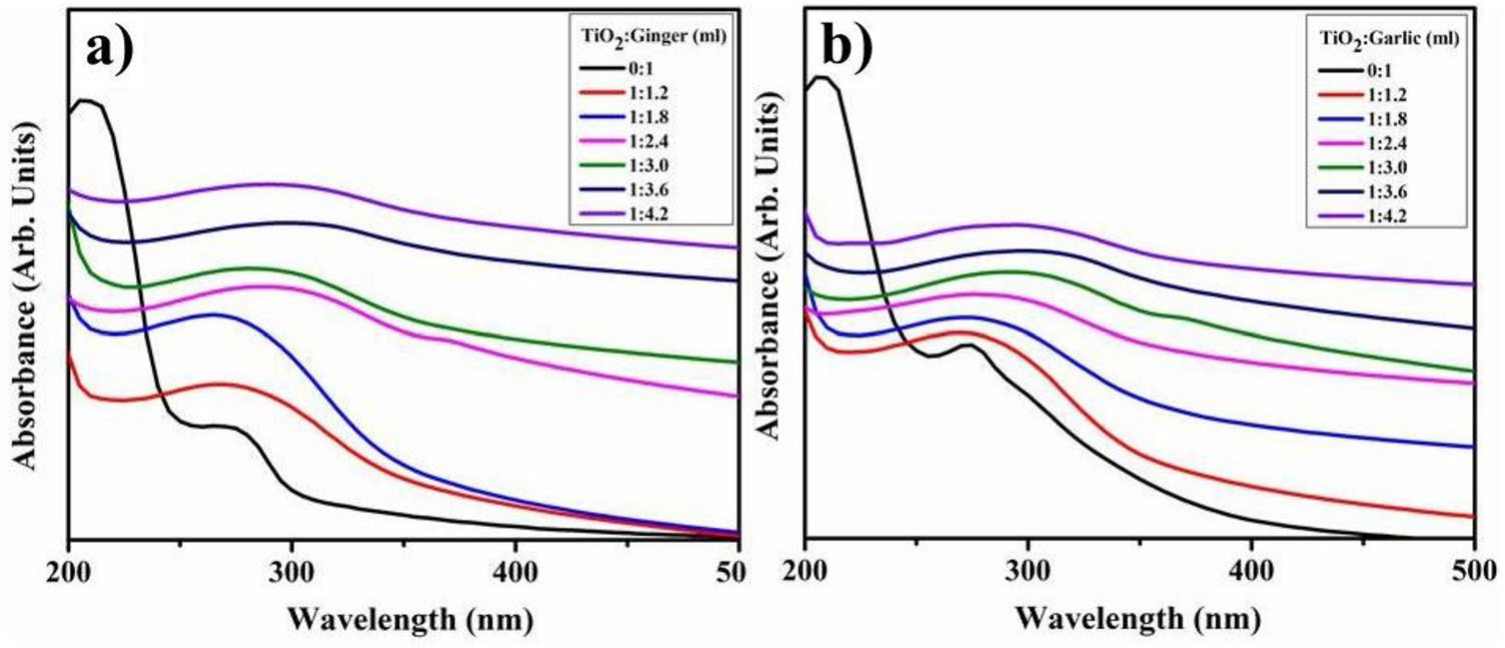
Absorption spectra of biosynthesized TiO_2_-NPs with ginger (**a**) and (**b**) garlic.

Visual attributes of ginger and garlic-reduced TiO_2_ between 200 and 500 nm are shown in Fig. 5a,b. Following a blueshift, the total absorbance (λ_max_) of TiO_2_-NPs measured at 318 nm (1:3.6 ml) increased with higher extract concentration. Ginger and garlic CAE absorption peaks were exhibited at 275 and 280 nm, correspondingly. The breadth of peak showed aggregation of particles, and the passage of electrons to conduction bands was indicated by the significant absorption. Consequently, Fig. 5a,b depicts a reduction in NP absorption with increasing or decreasing extract volumes beyond the optimal range (1:3.6 ml).

**Figure 6:**
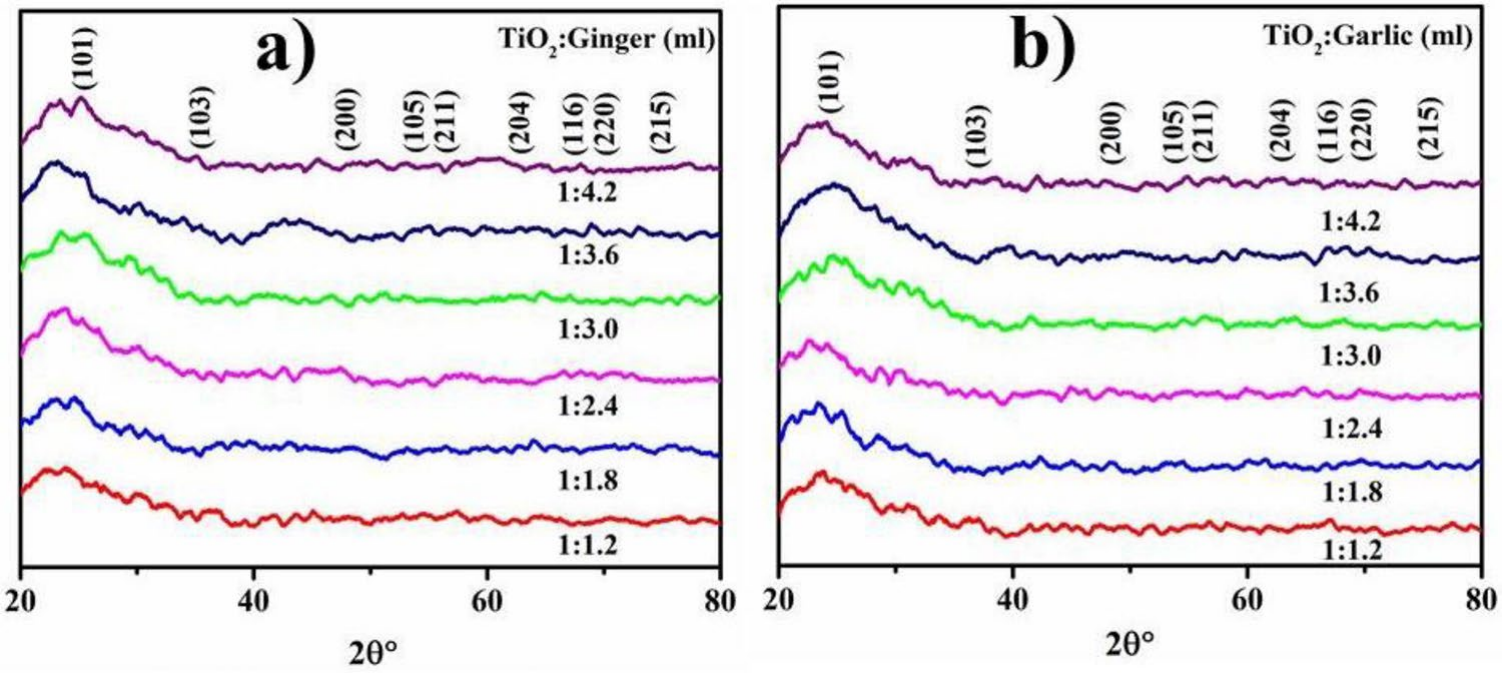
XRD pattern of fabricated TiO_2_ with ginger (**a**) and garlic CAE (**b**).

The crystalline geometries, phase development, and sizes of TiO_2_-NPs were studied with XRD as displayed in Fig. 6a,b. The highs at 2θ values of 25.28°, 36.94°, 48.05°, 53.89°, 55.06°, 62.69°, 68.76°, 70.31°, 75.03° match with (101), (103), (200), (105), (211), (204), (116), (220), and (215) planes (JCPDS card no. 00-021-1272) Fig. 6a,b. The peak intensity indicates tetragonal TiO_2_ with an average size of 23.38 nm, as determined using D = 0.9λ/βcosθ for ginger and 58.64 nm for garlic phytochemically-reduced TiO_2_-NP.

**Figure 7:**
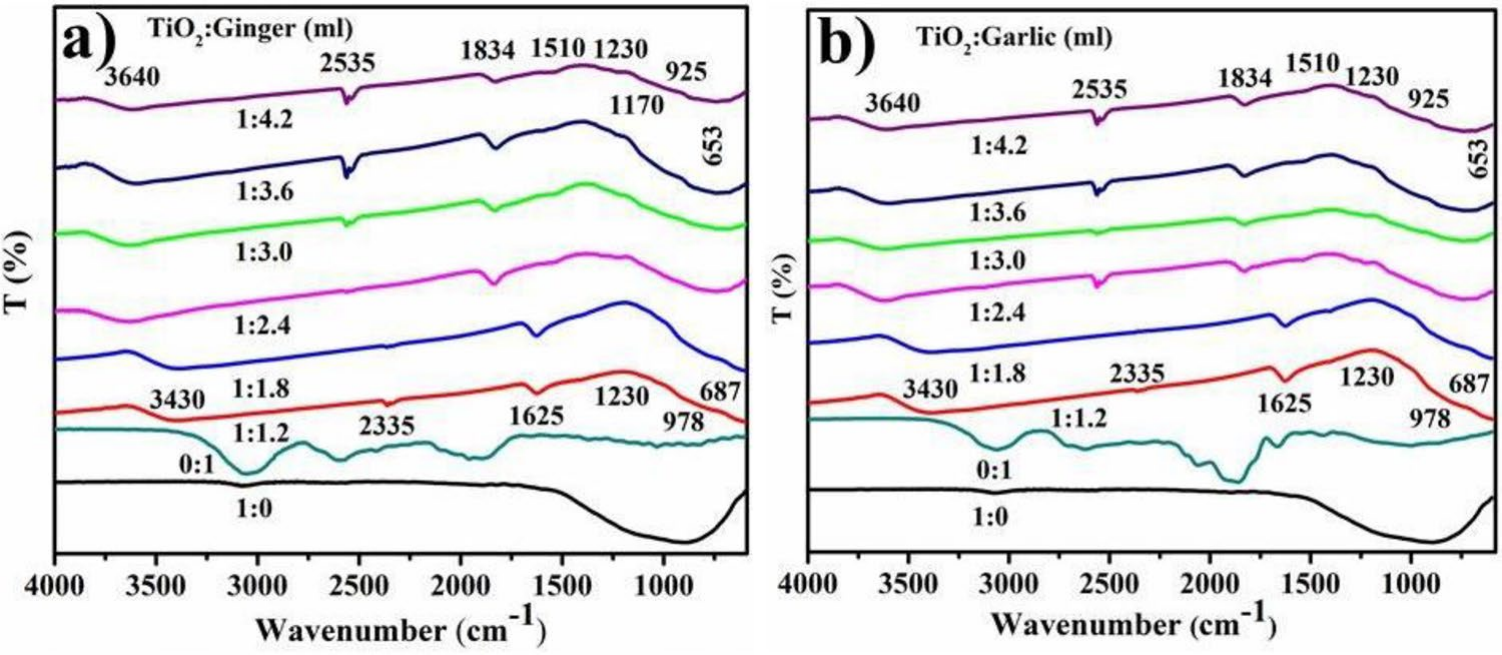
FTIR spectra of TiO_2_ reduced with ginger (**a**) and garlic CAE (**b**).

The FTIR spectrum of TiO_2_ produced using CAE of roots is shown in Fig. 7a,b as a broad absorption at 3640 cm^−1^ corresponding to OH and a carbonyl group with (N–H) amine stretching. Sharp absorptions at 3430 and 1625 cm^−1^ show the existence of water and hydroxyl groups. Absorption bands below 1200 cm^−1^ are indicative of Ti–O–Ti vibrations, whereas a high absorption spike at 2335 cm^−1^ confirms C≡N stretching. The absorption peak between 500 and 700 cm^−1^ corresponds to Ti–O stretch and Ti–O–Ti bridge stretching modes. The signal at 653 cm^−1^ suggested that phytochemically biologically-synthesized TiO_2_ anatase phase contribution was present. The significant peaks at 978 and 687 cm^−1^ correspond to the O–Ti–O bond and metal oxygen stretching frequency, correspondingly. After TiO_2_ reduction, changes in the peaks at 2335–2535 and 1625–1834 cm^−1^ were observed.

**Figure 8:**
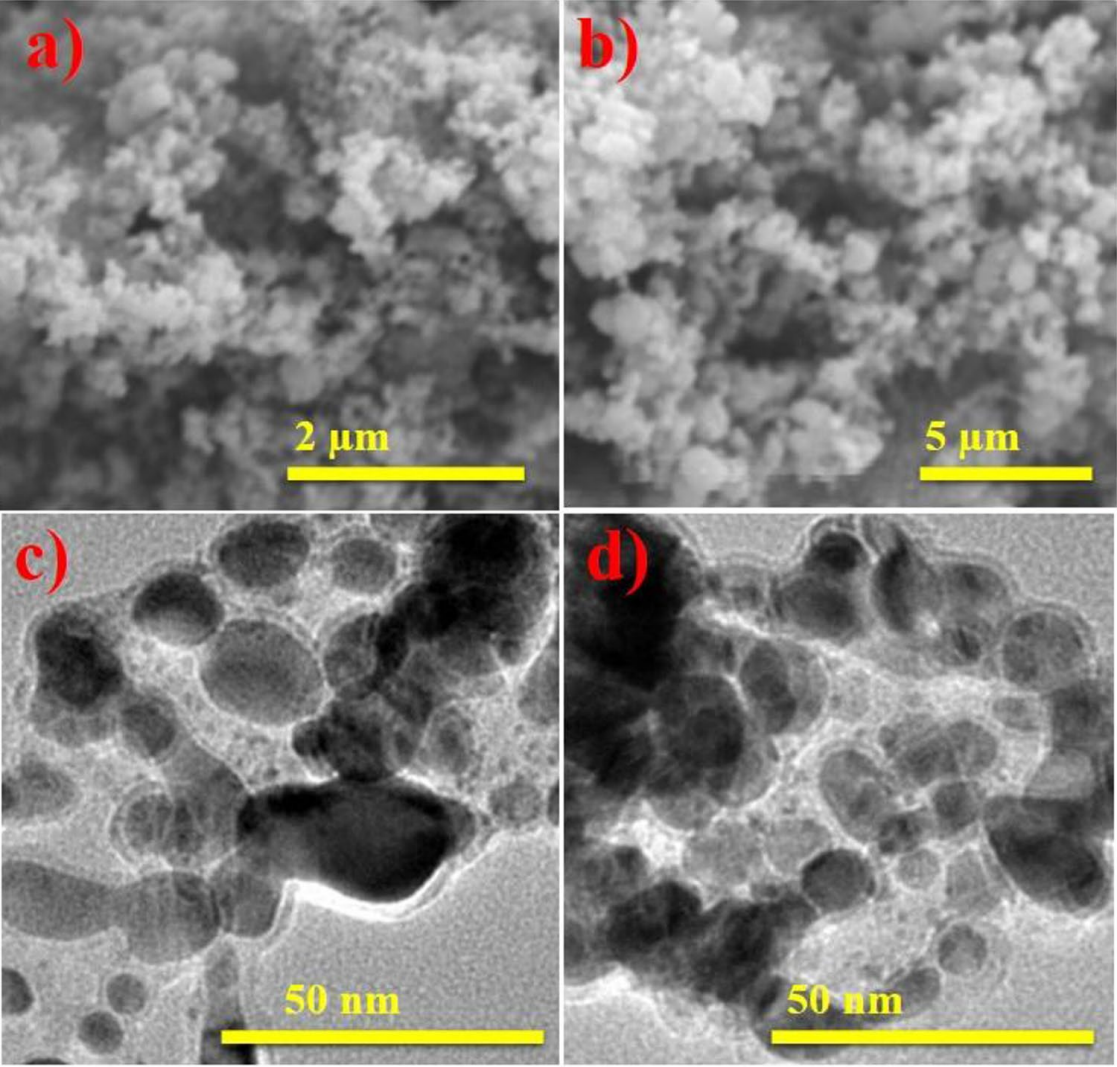
FE-SEM analysis of biologically-reduced TiO_2_ with ginger (**a**) garlic (**b**) TEM analysis of reduced TiO_2_ with ginger (**c**) and garlic (**d**).

As illustrated in Fig. 8a,d, the morphology and size of biologically-reduced TiO_2_-NPs were determined using scanning (FE-SEM) and transmission (TEM) electron microscopes. FE-SEM analysis revealed spherical topography with dense aggregation. The TiO_2_-NPs, TEM images displayed more spherical (< 50 nm) morphology with aggregation and indicated that increasing extract concentration may enhance aggregation.

**Figure 9:**
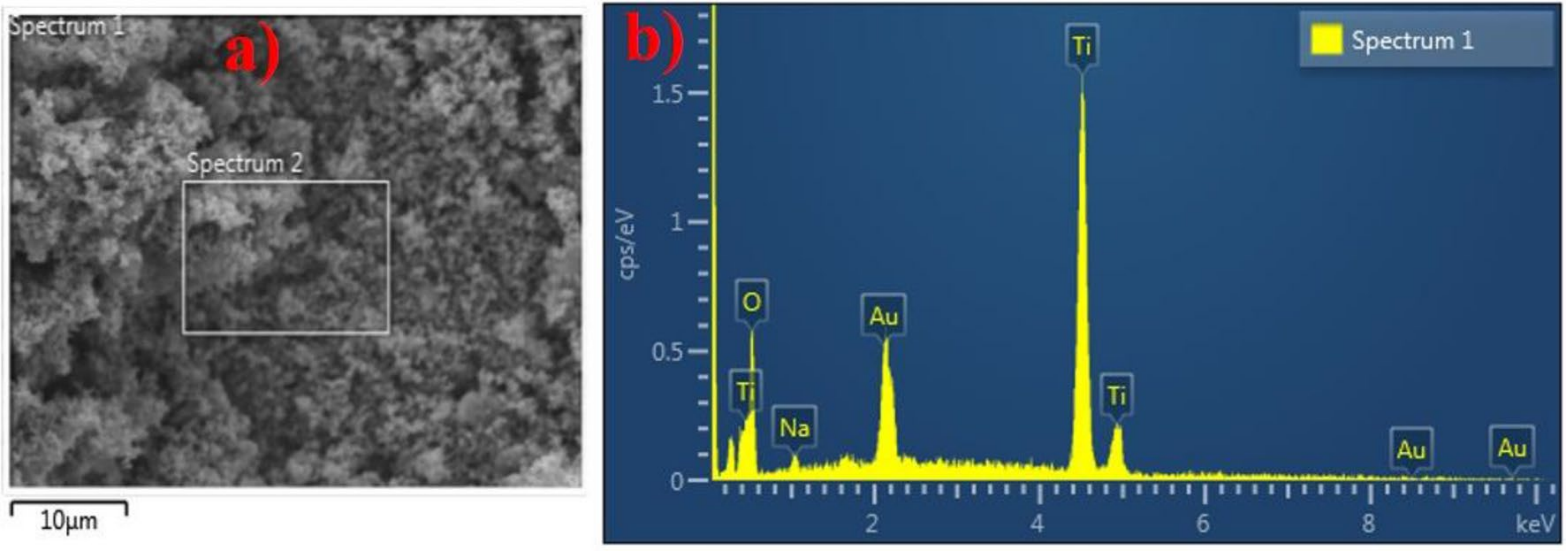
(**a**,**b**) SEM image and EDS spectrum of fabricated TiO_2_-NPs.

As illustrated in Fig. 9a,b, energy dispersive spectroscopy (EDS) was used to quantify the elemental composition of produced TiO_2_-NPs. The EDS spectrum of samples evaluated between 1 and 10 keV exhibited three peaks clearly connected to the high purity of Ti. Ti and O have respective atomic weight fractions of 81.1% and 18.3% as determined by their spectra. In addition, Au and Na peaks were detected, which were attributed to the sputtered coating, specimen holder, the conductive strip, contaminant and minerals found in roots extracts.

**Figure 10:**
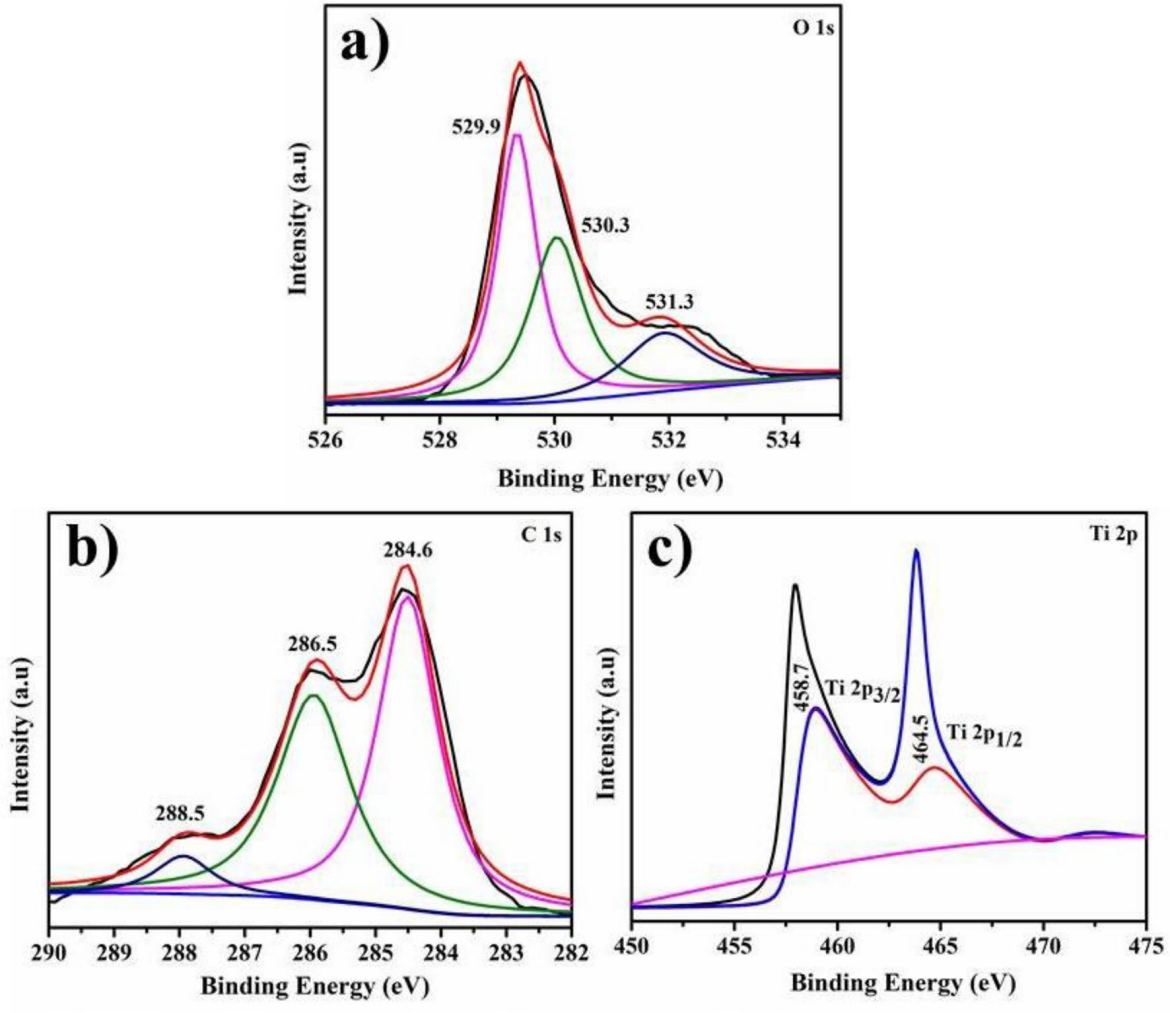
XPS spectra of doped particles O1s (**a**) C1s orbitals (**b**) Ti 2p spectra (**c**).

XPS shows O1s, C1s and Ti 2p spectra of biologically-reduced TiO_2_-NPs in Fig. 10a–c that suggests synthesized samples chemical nature and surface bonding states. The O1s peak at 529.9 eV Fig. 10a could be assigned to lattice oxygen atoms. The contribution located at 530.3 eV is ascribed to Ti_2_O_3_ and peak 531.3 eV is attributed to non-lattice oxygen. The most intense peak 284.6 is considered as reference peak for calibration of other binding energies in C 1 s spectrum Fig. 10b. Peaks appearing at 286.5 and 288.5 eV correspond with C–O and C=O bonds, respectively. The Ti 2p spectrum containing Ti 2p_3/2_ and Ti 2p_1/2_ peaks at B.E 458.7 and 464.5 eV, respectively depict typical characteristic of Ti^4+^ –O bond of TiO_2_ Fig. 10c.

**Figure 11:**
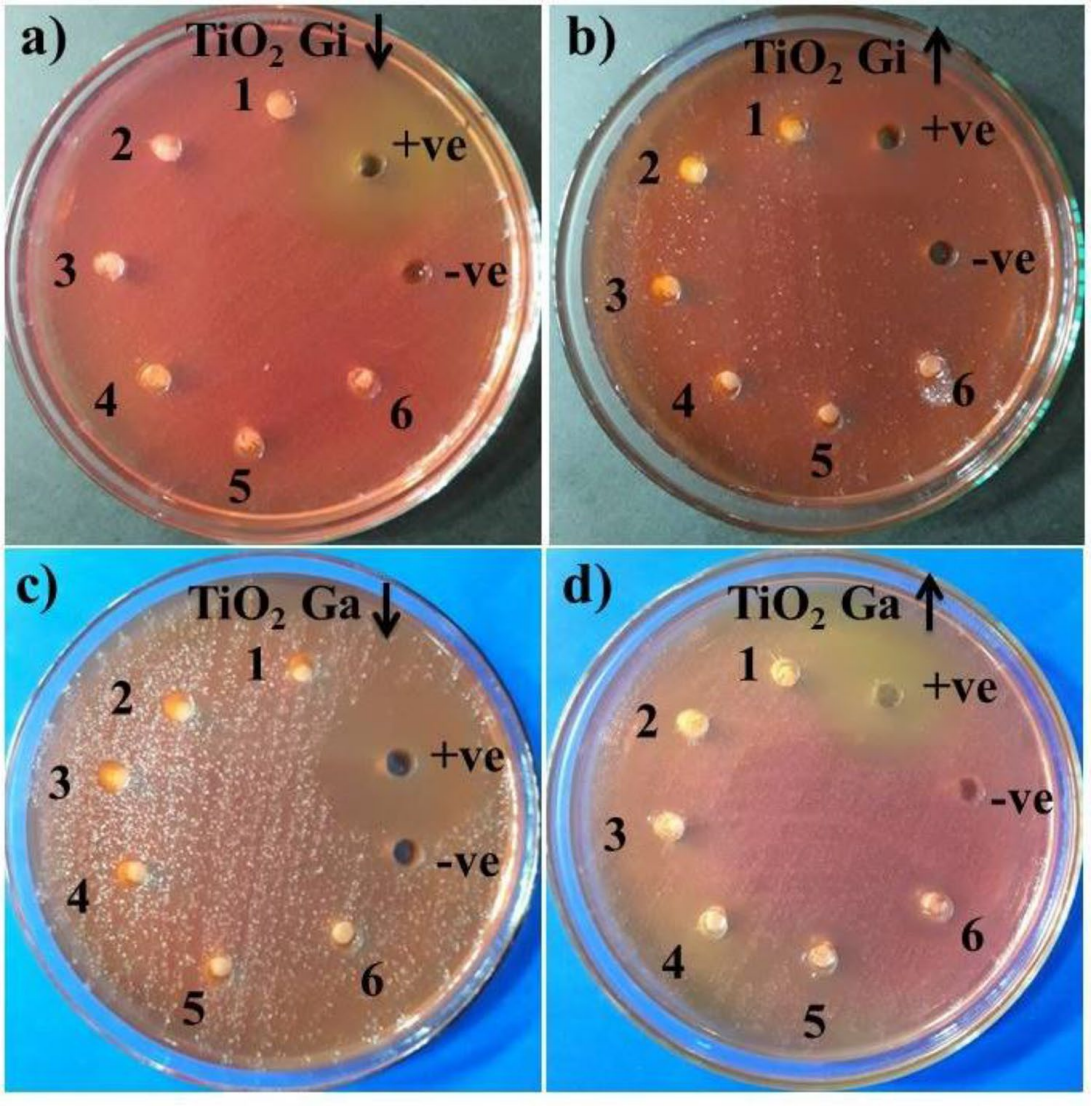
*In-vitro* bactericidal potential of biologically-reduced TiO_2_-NPs with ginger CAE at the minimum and maximum concentrations (**a**, **b**) garlic (**c**, **d**).

Well diffusion technique by estimating inhibition areas (mm) as seen in the Fig. 11a–d and Table 1, tested the microbicidal potential of ginger and garlic roots CAE and TiO_2_-NPs. The comparative analysis with published literature is depicted in Table S1. The results revealed a strong relationship between concentration and inhibitory regions (mm). Statistically significant inhibition regions (mm) (*p* < 0.05) were exhibited for samples 1 (1.2 ml:1), 2 (1.8 ml:1), 3 (2.4 ml:1), 4 (3 ml:1), 5 (3.6 ml:1) and 6 (4.2 ml:1) with a range of (0.95–1.55 mm) and (1.05–2.65 mm) at the minimum and maximum concentrations for ginger CAE reduced TiO_2_-NPs as seen in Fig. 11a,b while, (1.40–3.55 mm) for NPs that were biologically reduced with garlic CAE against MDR *S. aureus* as seen in Fig. 11c,d. TiO_2_ biologically-reduced with garlic root CAE depicted null activity. Both findings were compared with –ve DIW (0 mm) and + ve ciprofloxacin (7.50 mm) controls. In conclusion, garlic-reduced TiO_2_-NPs at elevated concentration exhibited significantly (*P* < 0.05) improved antibacterial activity against MDR *S. aureus*.

**Table 1:** Bactericidal efficacy of TiO_2_-NPs.

Microorganism	Sample	^a^Inhibition region (mm)		^b^Inhibition region (mm)	
		500 μg/50 μl	1000 μg/50 μl	500 μg/50 μl	1000 μg/50 μl
MDR *S. aureus*	(1.2 ml:1) 1	0	1.05	0	1.4
	(1.8 ml:1) 2	0	1.35	0	1.95
	(2.4 ml:1) 3	0	1.5	0	2.35
	(3.0 ml:1) 4	1.15	2.05	0	2.85
	(3.6 ml:1) 5	1.55	2.65	0	3.55
	(4.2 ml:1) 6	0.95	2.35	0	3.25
	Ciprofloxacin	7.5	7.5	7.5	7.5
	DIW	0	0	0	0

^a^Inhibition areas of synthesized TiO_2_ from ginger CAE.

^b^Inhibition areas measurements (mm) of NPs incorporated by garlic.

**Figure 12:**
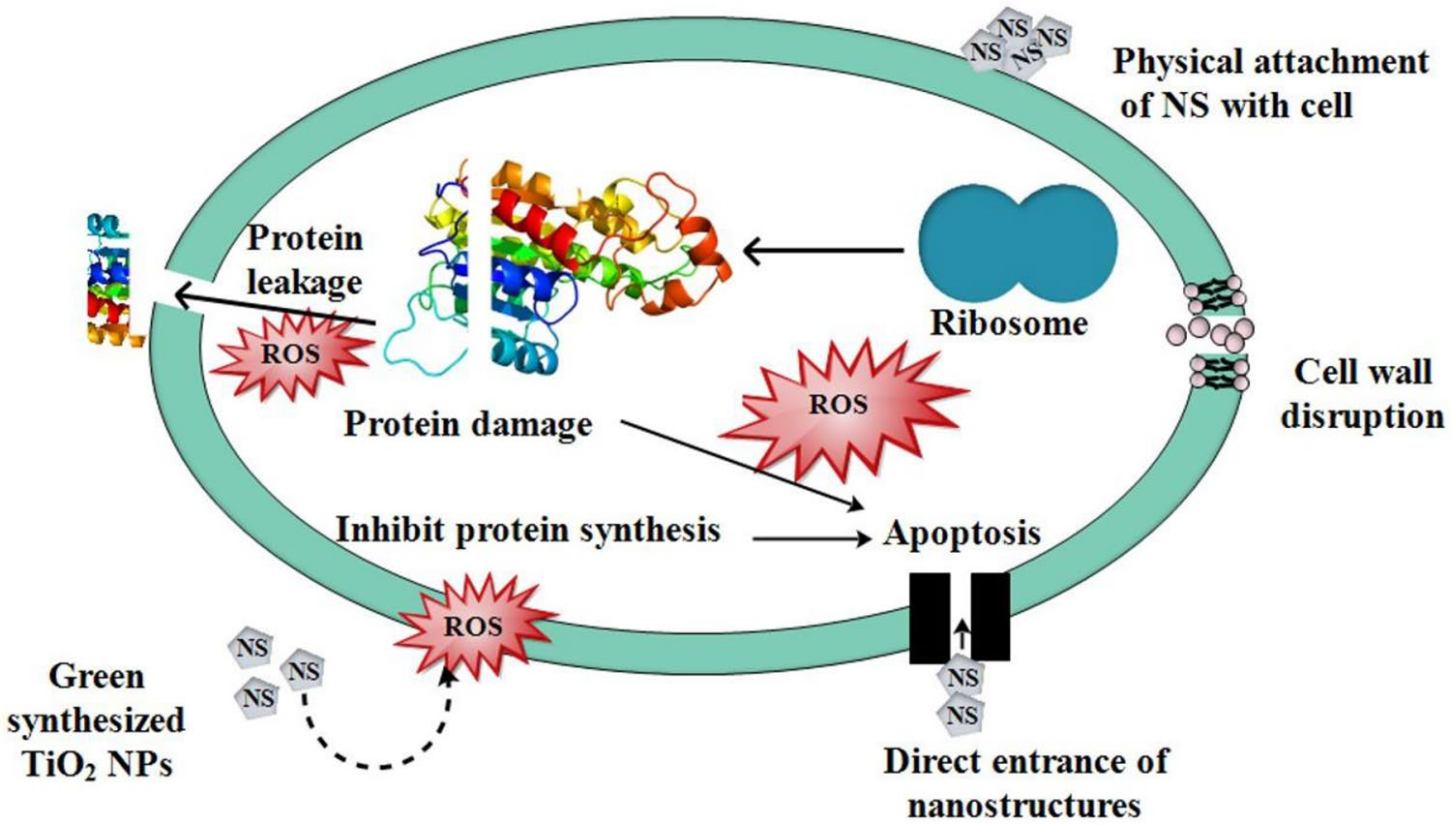
Mechanistic illustration of antibacterial activity of garlic and ginger doped TiO_2_ NPs.

The oxidative destruction induced by NPs is dependent upon their morphology, concentration, and scale, which are inversely attributed to the doped substance's characteristics^65^. The reactive oxygen species (ROS) generated by NPs persist effectively inside the pathogen cell membrane, causing cytoplasmic expulsion and pathogen violence as presented in Fig. 12^66^. Strong cationic interaction of Ti^4+^ at greater concentrations with the negative virulent organism causes cellular degeneration and destruction of bacteria^67^.

**Figure 13:**
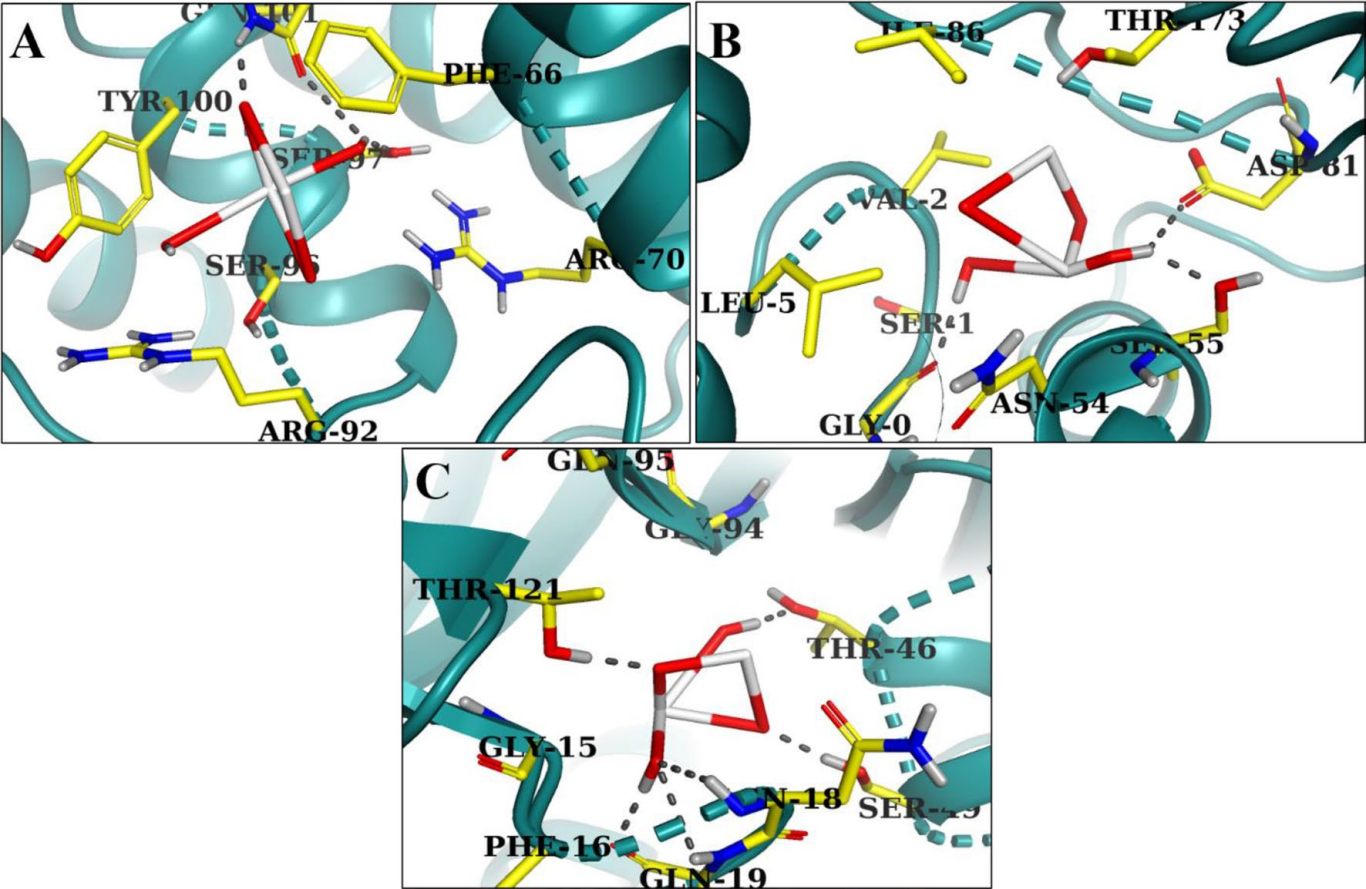
Binding interaction pattern of TiO_2_ nanoparticles with active site residues of (**A**) Thymidylate kinase, (**B**) DNA gyrase and (**C**) Dihydrofolate reductase from *S. aureus*.

To gain insight into the mechanism of interactions between TiO_2_ nanoparticles and target enzymes, a molecular docking study was conducted. Folate biosynthetic process results in the production of tetra-hydrofolate, which is required for biosynthesis of several bioactive elements, including thymidylate enzyme, pan-tothenic acid, nitrogenous bases as purine, ribonucleic acid, and amino acids. Dihydrofolate reductase and thymidylate kinase, enzymes related to such pathway, have been suggested as interesting candidates for antibiotic development^68,69^. Keeping in mind the significance and need of folate biosynthesis pathway for the development and survival of bacteria, the binding interface pattern of such nanoparticles was examined against *S. aureus* DHFR, TMK, and DNA gyrase enzymes. The best-docked shape of TiO_2_ nanoparticle into active pocket of DHFR has a binding energy of 4.57. As demonstrated in Fig. 13C, TiO_2_ is linked with THR121, Thr46, Ser49, Asn18, and Gln19 through H-bonding. Additionally, in the case of TMK, the highest binding score of 4.73 is attributed to H-bonding interactions with Ser97 and Gln101, as shown in Fig. 13A–C.

As illustrated in Fig. 13B, TiO_2_ nanoparticles exhibited H-bonding interactions with Gly0, Asp81, and Ser55 in case of *S. aureus* DNA gyrase through binding score of 4.40. Table 2 provides a summary of docking values and critical residues implicated in H-bonding for all proteins. The considerable binding score and interaction of TiO_2_ nanoparticles revealed that they are a potential blocker of dihydrofolate reductase, TMK, and DNA gyrase, whose inhibitory potential can be further investigated.

**Table 2:** Surflex score of docked ligand TiO_2_.

Proteins	CScore^a^	Crash score^b^	Polar score^c^	G score^d^	PMF score^e^	D score^f^	Chem score^g^	Amino acid interaction
DHFR	4.57	− 0.01	4.65	− 27.870	8.905	− 307.623	− 3.695	T121, T46, S49, N18, Q19
TMK	4.73	− 0.06	5.62	− 77.258	− 4.599	− 155.269	− 10.337	S97, Q101
GyrB	4.40	− 0.11	5.09	− 22.486	13.091	− 70.818	− 3.695	G0, D81, S55

^a^CScore is a consensus scoring system that ranks the attraction of ligands based on different scoring functions.

^b^Crash-score indicating improper binding site piercing.

^c^Polar domain of ligand.

^d^G-score representing hydrogen bonding, complex (ligand–protein), and internal (ligand–ligand) energies.

^e^PMF-score showing Helmholtz free energies for protein–ligand atom pairings (Potential of Mean Force, PMF).

^f^D-score for charge and van der Waals interactions among protein and ligand.

^g^Chem-score in addition to an intercept term points are awarded for hydrogen bonding, lipophilic contact, and rotational flexibility.

**Figure 14:**
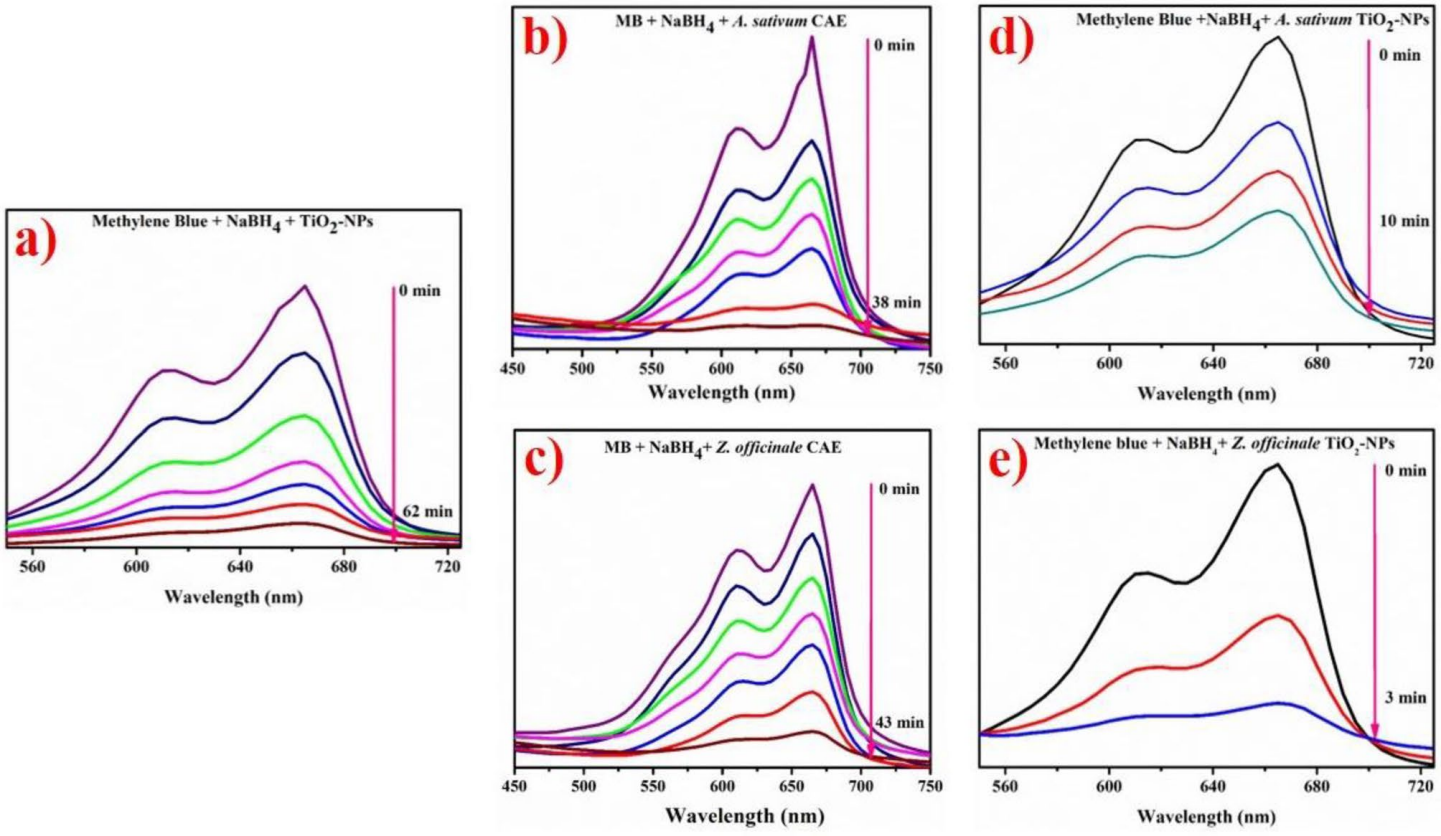
Catalytic reactivity of TiO_2_ (**a**) garlic CAE (**b**) ginger CAE (**c**) biologically reduced TiO_2_ with garlic CAE (**d**) and ginger doped NPs (**e**).

Figure 14a–e illustrates the substantial decrease in catalytic MB at room temperature with roots CAE and biologically reduced TiO_2_-NPs. Figure 14() demonstrates the catalytic activity of standard TiO_2_-NPs purchased from Sigma Aldrich. Figure 14b,d indicates the catalytic potential of garlic CAE and reduced TiO_2_-NPs with garlic CAE. Figure 14c,e depicts the catalytic activity of CAE of ginger and doped TiO_2_-NPs with ginger CAE. Generally, TiO_2_ and CAE of garlic and ginger roots diminished MB in 62, 38, and 43 min, respectively (see Fig. 14a,b, and e) compared to a time of 10 min and 100% reduction of dye in 3 min for garlic and ginger CAE doped TiO_2_ NPs, as presented in Fig. 14d,e.

**Figure 15:**
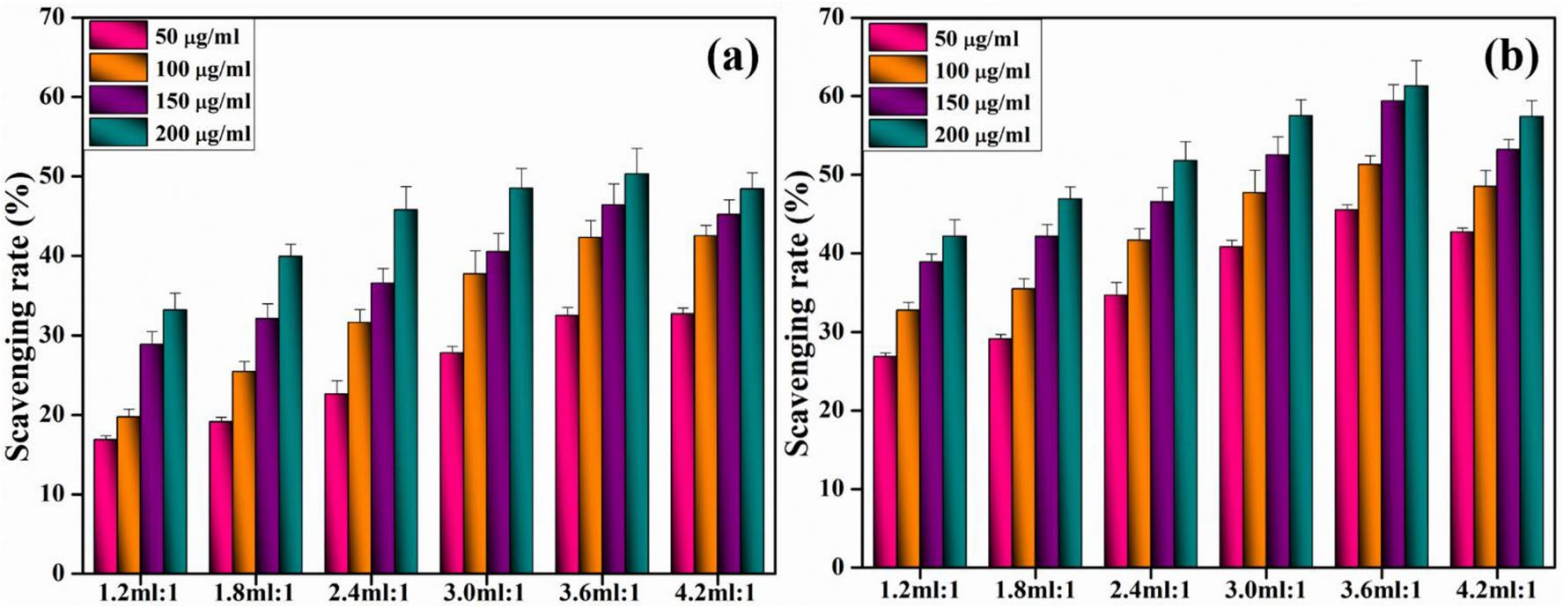
Scavenging potential of synthesized TiO_2_-NPs (**a**) ginger and (**b**) garlic.

The DPPH scavenging experiment in Fig. 15 is used to evaluate and quantify anti-oxidant capabilities of scavenging DPPH radicals, which are active radical species. Anti-oxidative properties of compounds are linked to their capacity to donate hydrogen or electrons to DPPH free radicals, leading to diamagnetic compounds with high stability^70^. All compounds exhibited anti-oxidant potential that scaled with increasing doses. TiO_2_-NPs with maximum ginger and garlic concentrations inhibited DPPH radicals by donating hydrogen atoms at concentrations of 200 g/mL (50.3% for ginger and 61.4% for garlic) in 3.6 ml: 1 sample whereas, turbidity of sample may be responsible for the modest drop in 4.2 ml: 1 sample^71^.

## Discussion

In the present study, TiO_2_ peak width in UV–Vis indicated agglomeration of particles and electronic transition towards conduction bands is shown by strong absorption^72^. In XRD, the peaks at 2θ values of 25.28°, 36.94°, 48.05°, 53.89°, 55.06°, 62.69°, 68.76°, 70.31°, 75.03° correspond to (101), (103), (200), (105), (211), (204), (116), (220) and (215) planes (JCPDS card no: 00-021-1272)^73^. The large peaks demonstrate oxygen presence^74^. The FTIR analysis of TiO_2_ synthesized with roots extracts, as seen in Fig. 7a,b, showed wide absorption at 3640 cm^−1^, which corresponds to the presence of OH while the peak width validated the presence of carbonyl group with (N–H) amine stretching^75^. Absorption peaks below 1200 cm^−1^ depicted Ti–O–Ti vibrations while, intense peak at 2335 cm^−1^ confirmed C≡N stretching^73^. The absorption peak at 500–700 cm^−1^ corresponded to Ti–O stretching and bridging-stretching modes of Ti–O–Ti^76,77^. The peak present at 653 cm^−1^ indicated contribution from phytochemically synthesized TiO_2_ anatase phase^78^. Peaks at 978 and 687 cm^−1^ prominently corresponded to O–Ti–O bond and metal oxygen stretching frequency^79,80^. After TiO_2_ reduction, peak changes were observed at 2335–2535 and 1625–1834 cm^−1^ indicating presence of phytochemicals, flavonoids, proteins containing ketones, carboxylic acid and amines that are considered significant for reduction^81^. The TiO_2_-NPs showed pleomorphism during FE-SEM examination with exhibition of cubical and spherical morphology (< 50 nm) accompanied by increased agglomeration^82^.

Agglomeration of NPs suggests polymer conformity and the presence of magnetic forces between particles^83^. In XPS analysis, the O1s contribution located at 530.3 eV is ascribed to Ti_2_O_3_ and the peak at 531.3 eV is attributed to non-lattice oxygen^84,85^. Peaks appearing at 286.5 and 288.5 eV corresponded with C–O and C=O bonds, respectively^86,87^. The Ti 2p spectrum containing Ti 2p_3/2_ and Ti 2p_1/2_ peaks at B.E 458.7 and 464.5 eV, respectively depict typical characteristic of Ti^4+^ –O bond of TiO_2_^88,89^. The production of highly reactive ^·^OH and ^·^O_2_ radical species can interact with DPPH free radicals, triggering the breakdown of DPPH, which is intrinsically linked to standard ascorbic acid^90^.

The primary challenge in producing NPs sustainably has been in regulating their final dimensions and forms. Various phytochemical constitutions in plants have specific molecular parameters, and their forms and dimensions are reflected accordingly. Furthermore, the chemical makeup of similar plants cultivated in diverse regions or harvested at distinct times of year might also lead to differences in active ingredients. As a result, the diameter and form of the precipitated NPs would be affected. These might also reduce their worth in the market, since commercialized nanoparticles are often well-suited to their intended application. As a result, it might be even more challenging to identify suitable uses and marketplaces for phyto-based NPs. When contrasted with preparing solutions employed in chemical procedures, botanical extracts have a far higher concentration of active compounds. Researchers are encouraged to further refine their methods for producing phyto-based NPs since the benefits are seen to outweigh the drawbacks. To get a deeper comprehension of the processes involved in the manufacturing and use of TiO_2_ NPs, the following future studies are recommended:

- Substantial tuning is needed to produce TiO_2_ NPs with appropriate morphology and size using a green production approach.
- The metabolites in botanical extracts ought to be analyzed further to identify their effectiveness regarding NPs production.
- The mechanical characteristic of TiO_2_ NPs generated using a green technique needs further research.
- Further research is required to determine the durability of TiO_2_ NPs generated using a green synthesis technique.

## Conclusion

This is the first research to quantify the bactericidal susceptibility of doped TiO_2_ nanostructures against MDR *S. aureus* from bovine mastitis. Incorporating ginger and garlic roots CAE in varying proportions had significant impact on the development and optimization of metal oxide nanostructures. Analysis by means of X-ray diffraction confirmed the presence of tetragonal TiO_2_. Biologically-reduced TiO_2_ with ginger and garlic CAE indicated crystallite sizes between 23.38 and 58.64 nm, as measured with XRD. The NPs exhibited pleomorphism and spherical morphology with dense aggregation, as determined with FE-SEM and TEM investigations. UV–Vis spectroscopy revealed an upsurge in absorbance as the quantity of extract in TiO_2_ increased, accompanied by a blueshift. The experimental findings demonstrated that extract-doped NPs are more effective catalysts compared to conventional NPs and CAEs alone. Compared to biologically-reduced NPs with ginger, the garlic CAE doped nanoparticles displayed improved bactericidal activity against MDR *S. aureus*. This research indicated that the emergence of antibiotic resistance might be considerably mitigated by using green fabricated metal oxide nanostructures as alternatives for antibiotic treatments. Molecular documentation, toxicological investigations and in-vivo efficacy tests that target infectious and resistant Gram-negative pathogenic microbes ought to be the primary concern of future investigations that address uses of green synthesized metal oxide NPs produced with economical and non-toxic green methods.

### Supplementary Information

Supplementary Information.

## Data Availability

The datasets used and/or analyzed during the current study available from the corresponding author on reasonable request.
