# Supplementary material for: Using biologically synthesized TiO2 nanoparticles as potential remedy against multiple drug resistant Staphylococcus aureus of bovine mastitis
Source: Sci Rep. 2023 Nov 1;13:18785. doi: 10.1038/s41598-023-45762-4 (PMC10620395; doi:10.1038/s41598-023-45762-4)
Supplement: Supplementary file 1 — Supplementary Information. [file 41598_2023_45762_MOESM1_ESM.docx]

**Using biologically synthesized TiO_2_ nanoparticles as potential remedy against multiple drug resistant *Staphylococcus aureus* of bovine mastitis**

Anwar Ul-Hamid*^1,2^, Nadeem Baig^3^, Ali Haider^4^, Abbas S. Hakeem^5^, and Muhammad Ikram^6^

^1^Core Research Facilities, King Fahd University of Petroleum & Minerals, Dhahran 31261, Saudi Arabia.

^2^Interdisciplinary Research Center for Advanced Materials, King Fahd University of Petroleum & Minerals, Dhahran 31261, Saudi Arabia.

^3^Interdisciplinary Research Center for Membranes and Water Security, King Fahd University of Petroleum and Minerals, Dhahran, 31261, Saudi Arabia.

^4^Department of Clinical Sciences, Faculty of Veterinary and Animal Sciences, Muhammad Nawaz Shareef University of Agriculture (MNSUA) Multan, 66000, Pakistan.

^5^Interdisciplinary Research Center for Hydrogen and Energy Storage, King Fahd University of Petroleum & Minerals, Dhahran 31261, Saudi Arabia.

^6^Solar Cell Applications Research Lab, Department of Physics, Government College University Lahore 54000, Pakistan.

*Correspondence: author email: anwar@kfupm.edu.sa

| **Table S1. Literature review comparison** | | | | | | |
| --- | --- | --- | --- | --- | --- | --- |
| **Material** | **Plant used** | **Pathogen** | **Concentration** | **Zone of**  **inhibition** | **Method** | **Ref** |
|  |  |  | **(mg mL^−1^)** | **(mm)** |  |  |
| TiO_2_ NPs | *Acorus calamus* | *S. aureus* | 0.01 | 10 ± 0.3 | Disk Diffusion | [1] |
|  |  | (ATCC 25923) | 0.02 | 12 ± 0.3 |  |  |
| TiO_2_ NPs | *Hibiscus flower* | *S. aureus* | 0.005 | 7 | Disk Diffusion | [2] |
|  |  |  | 0.01 | 12 |  |  |
|  |  |  | 0.015 | 13.5 |  |  |
|  |  |  | 0.02 | 14.5 |  |  |
| TiO_2_ NPs | *Mentha arvensis* | *S. aureus* | 10 | 0 | Disk Diffusion | [3] |
|  |  |  | 20 | 0 |  |  |
|  |  |  | 30 | 0 |  |  |
| TiO_2_  NPs | *Trigonella foenum-graecum* | *S. aureus* | 10 | 11.2 ± 0.4 | Disk Diffusion | [4] |
| TiO_2_ NPs | *Azadirachta indica* | *S. aureus* | 0.066 | 14.3 ± 1.46 | well diffusion | [5] |
|  |  |  | 0.133 | 17 ± 1.16 |  |  |
|  |  |  | 0.2 | 19.33 ± 1.45 |  |  |
| TiO_2_ NPs | *Zingiber officinale* | MDR *S. aureus* | 0.5 mg/50 µl | 1.55 | well diffusion | Present |
|  |  |  | 1.0 mg/50 µl | 2.65 |  |  |
|  | *Allium sativum* |  | 1.0 mg/50 µl | 3.55 |  | work |
| TiO_2_ NPs | *Artemisia haussknechtii* | MDR S.  aureus | 0.1 M | 0 | Disk Diffusion | [6] |
|  |  | ATCC 43300 | 0.01 M | 0 |  |  |
|  |  |  | 0.001 M | 0 |  |  |

**References**

[1] A. Ansari, V. Siddiqui, W. Rehman, M.A.- Catalysts, undefined 2022, Green Synthesis of TiO2 Nanoparticles Using Acorus calamus Leaf Extract and Evaluating Its Photocatalytic and In Vitro Antimicrobial Activity, Mdpi.Com. (2022). https://doi.org/10.3390/catal12020181.

[2] P. Kumar, A. Francis, T.D.-J.E. Nanotechnol, undefined 2014, Biosynthesized and chemically synthesized titania nanoparticles: comparative analysis of antibacterial activity, Researchgate.Net. 3 (2014) 2319–5541. https://doi.org/10.13074/jent.2014.09.143098.

[3] W. Ahmad, K.K. Jaiswal, S. Soni, Green synthesis of titanium dioxide (TiO 2 ) nanoparticles by using Mentha arvensis leaves extract and its antimicrobial properties, Inorg. Nano-Metal Chem. 50 (2020) 1032–1038. https://doi.org/10.1080/24701556.2020.1732419.

[4] S. Subhapriya, P.G.-M. pathogenesis, undefined 2018, Green synthesis of titanium dioxide (TiO2) nanoparticles by Trigonella foenum-graecum extract and its antimicrobial properties, Elsevier. (n.d.). https://www.sciencedirect.com/science/article/pii/S0882401017317151 (accessed June 5, 2023).

[5] B. Thakur, A. Kumar, D.K.-S.A.J. of Botany, undefined 2019, Green synthesis of titanium dioxide nanoparticles using Azadirachta indica leaf extract and evaluation of their antibacterial activity, Elsevier. (n.d.). https://www.sciencedirect.com/science/article/pii/S0254629919307148 (accessed June 5, 2023).

[6] M. Alavi, N. Karimi, Characterization, antibacterial, total antioxidant, scavenging, reducing power and ion chelating activities of green synthesized silver, copper and titanium dioxide nanoparticles using Artemisia haussknechtii leaf extract, Artif. Cells, Nanomedicine Biotechnol. 46 (2018) 2066–2081. https://doi.org/10.1080/21691401.2017.1408121.
